# Recent Developments in the Application of Inorganic Nanomaterials and Nanosystems for the Protection of Cultural Heritage Organic Artifacts

**DOI:** 10.3390/nano12020207

**Published:** 2022-01-10

**Authors:** Toma Fistos, Irina Fierascu, Radu Claudiu Fierascu

**Affiliations:** 1 Emerging Nanotechnologies Group, National Institute for Research & Development in Chemistry and Petrochemistry—ICECHIM, 060021 Bucharest, Romania; toma.fistos@icechim.ro; 2 Department of Science and Engineering of Oxide Materials and Nanomaterials, University Politehnica of Bucharest, 011061 Bucharest, Romania; 3 University of Agronomic Sciences and Veterinary Medicine of Bucharest, 011464 Bucharest, Romania

**Keywords:** inorganic nanomaterials, cultural heritage, organic artifacts, protection, conservation

## Abstract

Cultural heritage (CH) represents human identity and evidence of the existence and activities that people have left over time. In response to the action of aggressive degrading factors, different materials have been developed and used to protect cultural heritage artifacts. The discovery of optimal materials for this purpose also raises several problems, mainly related to their compatibility with the support material, the most important aspect being that they must preserve their aesthetic characteristics. In this context, the present review paper aims to provide a critical discussion about the possibilities of using different inorganic nanomaterials and recipes for the conservation of cultural heritage objects of organic nature (such as paper, wood, and other support materials). In addition, also are covered different aspect concerning protection mechanisms and application methods as well as future perspectives in this area.

## 1. Introduction

Preservation of cultural heritage represents a forefront issue for researchers all over the world, as cultural heritage artifacts are continuously affected by a series of degradation factors, ranging from environmental aspects to natural or human-induced degradation [1].

In recent years, many scientific areas have been involved in the search for the best solutions to stop the degradation of cultural heritage and to preserve it without irreparably affecting its appearance. The search for an optimal conservation material also needs to consider its compatibility with the support material, as one of the most important properties required for such materials is that they must not alter the aesthetic characteristics of the treated object. Another problem that must be addressed in the development of these materials is the cost, which must be as low and accessible as possible, and the synthesis of these materials as simple as possible without many steps or obtaining many secondary compounds.

Over the last decades, perhaps one of the most important areas of research is represented by the field of nanomaterials and nanotechnology [2]. The term “nanomaterial” may refer to materials composed of single elements, such as metals or carbon, or materials composed of several elements, such as metal oxides or composites, with nano dimensions. The ability to manipulate and control materials at the atomic level and the subsequent understanding of fundamental processes at the nanoscale have led to new challenges. The reason for this is based on the unique and sometimes unexpected physical and chemical properties that are present in nano-level materials such as the increased surface-to-mass ratio, diffusivity, and electrical, optical, and thermal properties. However, the application of nanoparticles (NPs) in the field of heritage conservation requires a broader approach combining materials science, petrophysics, microbiology, and cultural heritage conservation with many other scientific disciplines.

The advantages of developing and using materials at the nanoscale include the physico-chemical compatibility of inorganic nanomaterials with the support material; the reactivity and penetration capacity of a reinforcing product into the support material and, consequently, its effectiveness are potentially increased when its particle size is reduced to nano-dimensions; an increase in area in relation to the volume and larger surface, improving the electronic and optical properties and the chemical reactivity between the consolidating material and the support material, as a higher proportion of atoms is found on the surface compared to those inside [3,4].

The continuous progress in nanotechnology has led over the last years to the evaluation and proposal of new alternatives for the protection of artifacts of organic nature such as archaeological wood, paper, textile, and leather. Although not as commonly encountered, such as for the conservation of inorganic artifacts, the use of inorganic nanomaterials for the conservation of cultural heritage artifacts of organic nature represents an emerging field of research [5].

In recent years, several review works have been published in the area of cultural heritage preservation using nanomaterials, focusing either on the topical application of selected nanomaterials in the conservation of cultural heritage objects [6], protection of certain types of support materials [7,8,9], or on the development of antimicrobial agents for cultural heritage application [10]. Although of certain scientific value, these works do not sufficiently cover the area of organic artifact protection and how nanotechnology can contribute to obtaining superior recipes.

In this context, the present review paper aimed to present a critical discussion regarding the possibilities for the use of different materials and recipes for the conservation of cultural heritage objects of organic nature (i.e., paper, wood, and textile). Different aspects about protection mechanisms and application methods are also covered.

## 2. Cultural Heritage Objects of an Organic Nature

Generally speaking, artifact materials can be classified based on standard typologies according to the used materials and manufacturing techniques. One of the main categories is the class of organic artifacts, where the typology includes a variety of objects made from organic materials such as wood, plant fibers, bone, antler, leather, ivory, and shell.

In a particular context, a classification is almost impossible to realize in a correct and complex manner. Baxter describes Read’s pioneering paper regarding artifact classification “as one of the best papers of its kind” [11], where a recursive subdivision can be made classifying the objects into obvious groups, and then sub-division of the groups/types can be made based on different variables or qualities of the artefacts studied.

Organic artifacts represent a very large portion of the objects currently found in museums all over the world, being considered extremely vulnerable to deterioration [12], as climate factors, pollution, or microbial attacks can lead to their degradation with specific impacts on each type of object.

The most encountered organic materials found in museum collections are represented by paper artifacts, textiles, leather, paintings on canvas, wood objects, or the so-called “ecofacts”, such as ivories or bones, containing both organic and inorganic matter [13].

**Figure 1:**
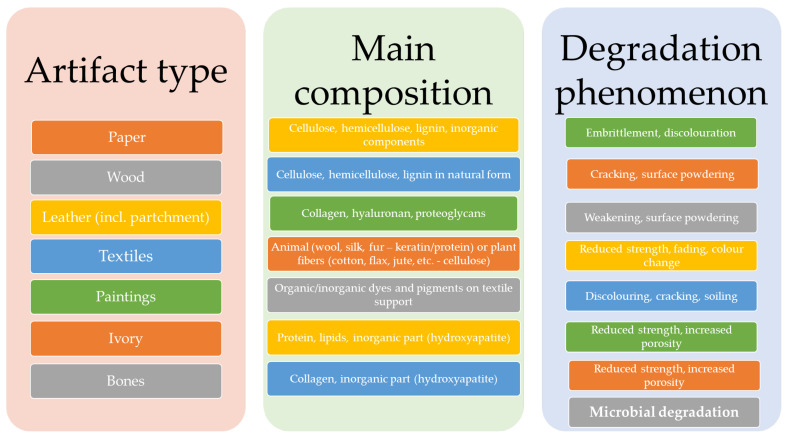
Main types of organic artifacts and their most encountered degradation phenomena.

Figure 1 presents the most encountered organic artifacts, their main composition, and the most encountered degradation phenomena.

In order to understand the potential application of nanomaterials for the protection of organic cultural heritage artifacts, some considerations related to the main factors affecting their longevity are necessary.

All artifacts of organic nature are affected by microbial degradation. Several types of bacteria and fungi have been identified on organic artifacts [14], which can lead to physical and chemical damage as well as aesthetic alterations. Cellulose-based artifacts are commonly affected by fungal species (*Ascomycetes*, *Aspergillus*, *Paecilomyces*, *Chrysosporium*, *Penicillium*, *Cladosporium*, and *Eurotium* or, in special cases, molds associated with water damage such as *Chaetomium*, *Monoascus*, *Epicoccum*, *Trichoderma*, and *Stachybotrys*). These organisms can produce staining of the artifact (i.e., the “foxing” phenomenon), induce embrittlement, or lead to the apparition of strong odors or toxic compounds through an enzymatic action [15]. Leather artifacts (including parchment) are commonly affected by species of the genera *Bacillus*, *Staphylococcus*, *Pseudomonas*, *Virgibacillus*, and *Micromonospora*; alkaliphilic bacteria (i.e., *Actinobacteria*); proteolytic fungi (i.e., *Chaetomium* and *Gymnoascus*); mitosporic fungi (i.e., *Acremonium*, *Aspergillus*, *Aureobasidium*, *Epicoccum*, *Trichoderma*, and *Verticillium*) associated with collagen biodeterioration [15]. Wood artifacts are affected by cellulolytic microorganisms (similar to paper artifacts) including cellulase-producing fungi (*Trichoderma*, *Fomitopsis*, *Aspergillus*, *Fusarium*, and *Neurospora*), brown rot (*Poria*, *Lenzites*, *Coniophora*, and *Tyromyces*) and white rot (*Phanerochaete*, *Sporotrichum*, and *Trametes)*, anaerobic cellulolytic fungi (*Neocallimastix*, *Piromyces*, and *Orpinomyces*), cellulolytic bacteria (*Bacillus*, *Acinetobacter*, *Cellulomonas*, and *Clostridium*), rumen bacteria (*Fibrobacter*, *Ruminococcus*, *Pseudomonas*, *Proteus*, and *Staphylococcus*), and thermophilic bacteria (*Anoxybacillus* and *Geobacillus*) [16]. A special case is represented by waterlogged wood artifacts. Wood artifacts in high-salinity marine environments are rapidly degraded by marine insects, while in low-salinity environments, decay occurs much slower. Moreover, artifacts found in marine sediments are mainly affected by erosion bacteria [17].

Biodegradation of collagen-based artifacts (leather and parchment) involves the chemical oxidative deterioration of amino-acid chains and hydrolytic cleavage of the peptide structure. The most encountered species belong to the genera *Bacillus, Staphylococcus, Pseudomonas*, *Virgibacillus*, and *Micromonospora*; alkaliphilic bacteria (i.e., *Actinobacteria*)*;* proteolytic fungi (*Chaetomium and Gymnoascus*); mitosporic fungi (*Acremonium*, *Aspergillus, Aureobasidium*, *Epicoccum*, *Trichoderma*, and *Verticillium*). The action of these microbial species can lead to the hydrolyzation of collagen fibers and other proteins or produce material discoloration [16].

Textile artifacts are affected by both microorganisms with cellulolytic and proteolytic activities, depending on the nature of the material. Commonly encountered microorganism on textiles of vegetal origin are fungi from the species *Alternaria*, *Aspergillus*, *Aureobasidium*, *Chaetomium*, *Cladosporium*, *Fusarium*, *Memnoniella*, *Mucor*, *Myrothecium*, *Paecilomyces*, *Penicillium*, *Rhizopus*, *Stachybotrys*, *Trichoderma*, *Trichothecium*, and *Verticillium* and bacteria belonging to the species *Arthrobacter*, *Bacillus*, *Cellulomonas*, *Cellvibrio*, *Clostridium*, *Cytophaga*, *Microbispora*, *Nocardia*, *Pseudomonas*, *Sporocytophaga*, and *Streptomyces*. Their action is mainly related to the enzymatic degradation of cellulose. Keratin-based textiles are affected by keratinolytic-inducing microorganisms (fungi—*Acremonium*, *Alternaria*, *Aspergillus*, *Cephalothecium*, *Chaetomium*, *Chrysosporium*, *Dematium*, *Fusarium*, *Microsporum*, *Oospora*, *Penicillium*, *Rhizopus*, *Scopulariopsis*, *Stachybotrys*, *Trichoderma*, *Trichophyton*, and *Ulocladium*; bacteria—*Alcaligenes*, *Bacillus*, *Proteus*, *Pseudomonas*, and *Streptomyces*), while textile containing mainly protein fibers (such as silk) are affected by microorganisms inducing proteolytic decomposition (fungi—*Aspergillus*, *Chaetomium*, *Cladosporium*, *Penicillium*, and *Rhizopus*; bacteria—*Aeromonas*, *Arthrobacter*, *Bacillus*, *Chryseomonas*, *Pseudomonas*, *Streptomyces*, *Serratia*, and *Variovorax*) [18]. 

Ivory and archeological bones are much less exposed to biodeterioration due to the high inorganic content (represented mainly by the hydroxyapatite). The microorganisms encountered are those associated with the degradation of proteins or collagen (as presented for other types of artifacts) or those related to the human skin microbiome or pathogenic bacteria and fungi (*Clostridiales* and *Phialosimplex*) or by opportunistic fungi [16].

Besides biodegradation, other deterioration processes are characteristic for each type of materials:

- Cellulose-based artifacts are subjected to acidic degradation of cellulose chains (due to the action of environmental or internal acids), alkaline degradation, photodegradation, and oxidative degradation;
- Collagen-based artifacts, under the action of light, elevated temperatures, humidity, and atmospheric pollutants undergo acidic hydrolysis or oxidative degradation of the functional side groups [19]. Under UV radiation, photodegradation of collagen into a powder form can also be encountered [20];
- Ivory and archaeological bones undergo similar degradation phenomena: while the collagen part of the artifacts can degrade by chemical hydrolysis [21,22], the degradation also affects the inorganic part, which can undergo mineral recrystallization and degradation of mechanical and morphological properties (such as increased porosity).

Nanomaterials can be used to counteract the effects of both biodeterioration as well as chemical or photodegradation, or it can even be used for the consolidation of the inorganic part of mixed artifacts. This can be achieved through harnessing their well-known properties (antimicrobial, UV protection, etc.) and selection of nanoparticles compatible with the support material. Moreover, some nanoparticles can react with the support material, leading to the formation of other compounds that can contribute to the consolidation of the artifacts.

## 3. Inorganic Nanomaterials for the Protection of Paper Artifacts

Paper artifacts represent one of the most fragile and, at the same time, widely spread cellulose-based objects. Compared with other similar objects, such as, for example, historical wood, paper artifacts possess particularities that make them more exposed to degradation including the treatment applied for paper manufacturing and their physical properties as well as the presence of inks and pigments on their surface. All these factors can lead to an accelerated degradation of paper artifacts for which the development of treatment methods represent an important research topic [23]. 

Considering the degradation processes commonly encountered in paper artifacts, the main application of nanomaterials as consolidants and preventive treatments are presented in Table 1.

**Figure 2:**
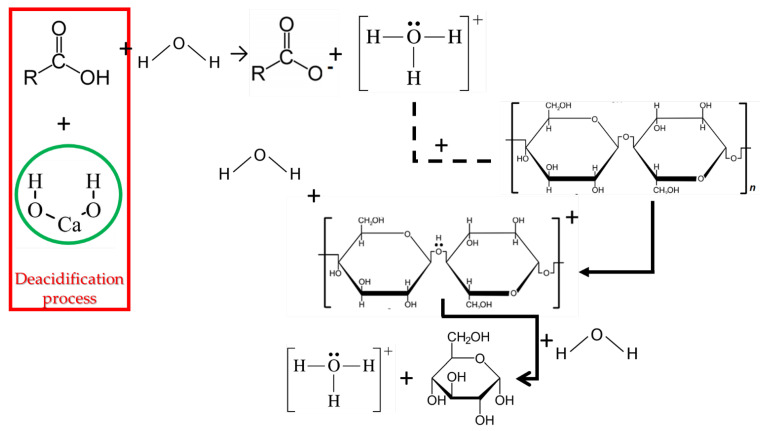
Acid degradation of cellulose, one of the most important degradation processes of paper artifacts, and the deacidification process (red square) realized by the addition of an alkaline reservoir (exemplified by calcium hydroxide).

The most important aspect regarding paper conservation is related to its deacidification. As already mentioned, acidification of cellulose-based objects in general (and of paper artifacts in special) leads to cellulose depolymerization (Figure 2); for paper objects, this has dramatic consequences, both in mechanical properties (the papers becoming brittle) and in the aesthetic characteristics (by darkening) [23,31].

Deacidification can be achieved by providing alkaline reservoirs; two main issues should be addressed when applying this strategy:

- Formulation of the deacidification solution, as the presence of some surfactants/stabilizers could lead to a reduction in nanoparticle reactivity, creating a too alkaline environment that could result in the alkaline depolymerization process [31];
- Compatibility of the proposed recipes with other elements present on the paper artifacts (such as inks, dyes, or pigments) [23].

In general, the deacidification of paper using metallic oxides occurs via the chemical interaction between the nanoparticles and acidic substances in the presence of CO_2_ by the conversion of oxides to hydroxides and carbonates [49]; metallic hydroxide act by reaction with atmospheric CO_2_, resulting in carbonate deposition (although they have as a main drawback the tendency to have values significantly higher than neutral) [50], while the carbonates acts as an “alkaline reserve buffer”, being able to neutralize the acidic species adsorbed onto the paper, or in situ generated, until they are exhausted [50].

Multiple alkaline solutions are presented in the literature (detailed in Table 1), mostly based on Mg or Ca oxides and hydroxides, as single components or incorporated in different treatment systems. As several commercial products are available on the market [37], we consider that the exhaustive presentation of these compounds is not necessary. The main conclusion that can be drawn from these studies is related to the possibilities to enhance these solutions, mainly in the nanomaterial’s synthesis stage. As emerging from the literature, the most promising morphology is represented by the nanosheets’ morphology, which allows for not only the development of the alkaline reserve, but it also covers the paper fibers, acting as lamination sheet, as a first line of defense against acid attack from environmental sources [33]. Calcium and sodium carbonates are also common apparition in literature studies as sources of alkaline reserve [42,46].

As an example of a less commonly used nanomaterial, Nemoykina et al. [40] presented the application of magnesium oxyhydroxide not only for the deacidification of an old low-quality book but also for its preservation. The developed lamellar nanostructures covered the paper fibers, strengthening them by surface bonding (25% increase), the samples becoming more resistant to aging. Even more, the deacidification treatment led to pH stabilization, as no significant decrease was observed upon aging and to a 10% increase in whiteness. 

Application of nanomaterials with known antimicrobial properties can provide protection against the biodeterioration induced by fungi or bacteria. This approach was presented by Jia et al. [35], who proposed the application of ZnO/nanocellulose composites as potent antimicrobial agents in the protection of paper artifacts. Their results showed that the nanocomposite not only provides a superior protection against biodeterioration, but it also possesses UV adsorption properties, inducing UV resistance as well as superior thermal resistance. 

A particular case (not presented in Table 1) is represented by the study of Hassan et al. [51], who applied ZnO (spherical, 21 nm) in hydroxypropyl cellulose for the reinforcement of papyrus samples. Although not technically a study performed on paper samples, we chose to present the study in this chapter, as the characteristics of papyrus are closely related to those of paper. The treatment led to a final pH of 7.08 (after artificial aging), a desired pH value for the conservation purposes, and to a conservation of tensile strength and elongation after the aging process. 

**Figure 3:**
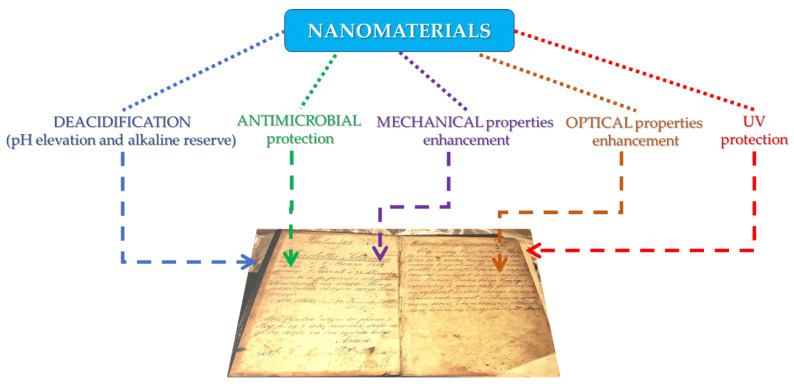
Application of nanomaterials for the protection of paper artifacts.

A schematic representation of the potential application of nanomaterials for the preservation of paper artifacts is presented in Figure 3.

## 4. Inorganic Nanomaterials for the Protection of Historical Wood

Wood represents a widely encountered material in the area of cultural heritage. Whether we are speaking of vernacular constructions, art objects, or ships, wood with historical value is encountered all over the world. Most of these objects are either part of outdoor constructions or waterlogged wood, as such being exposed to different environmental conditions which accelerates their decay. The treatment of degradation was traditionally performed by resection of the affected parts, followed by their replacement with other materials. This approach has currently been replaced with a more modern one, involving the preservation of the original material through consolidation as well as preventive treatment [52]. A common method for the treatment of historical wood involves the use of organic compounds (natural resins and bio-based or synthetic polymers), as recently presented by our group [53]. However, over the last years, new alternative treatments have been proposed. Among these consolidants and preventive treatments, several inorganic compounds can be identified in the literature (Table 2).

The use of nanoparticles in the area of historical wood preservation is focused on several main applications: consolidation of the wood samples (influencing the mechanical properties), deacidification (including neutralization of sulfur-containing acidic compounds), and antimicrobial protection. 

For example, Cavallaro et al. [61] presented the deacidifying consolidation of waterlogged archaeological woods using aqueous dispersions of polyethylene glycol (PEG) 1500 and halloysite nanotubes containing calcium hydroxide. By incorporating the calcium hydroxide into the halloysite nanotubes, a prolonged release was achieved, extending its deacidification action, recording a pH of 7.6 even 12 months after the treatment. Moreover, addition of the modified nanotubes to the polymer led to a remarkable increase in the mechanical performances in terms of flexural strength and rigidity compared with the pure PEG. 

Andriulo et al. [58] applied via direct dipping of calcium hydroxide nanoparticles obtained by the solvothermal method for the consolidation of waterlogged archaeological wood (softwood and hardwood), alum-treated archaeological wood (belonging to the Oseberg find), and sound oak. Their results revealed a pH increase from 2–3 to 5.5 for the waterlogged wood, an increase that was stable for over 1 month after treatment. Moreover, the treatment proved to be effective also in the case of very degraded waterlogged wood (for which the cellulose phase is almost completely destroyed, and a very high alum content is recorded).

Another example of the application of inorganic nanoparticles is represented by the study by Poggi et al. [54], which used calcium hydroxide nanoparticles obtained by solvothermal reaction for the deacidification of degraded waterlogged wood (oak wood specimens from original Vasa timber). By developing a specific vacuum treatment, the particles were used to treat the wood specimens, the pH and differential thermal fravimetric (DTG) measurements revealed that NP dispersions penetrated the wood, leading to its deacidification and the prevention of mechanical properties loss. 

Nanoparticles with well-known antimicrobial properties can be easily incorporated into polymeric matrixes or directly deposited onto the wood artifacts, allowing for the slow release of the NPs and a prolonged antimicrobial action as demonstrated by Harandi et al. [55], Ion et al. [62], and Yves et al. [68]. The application of UV adsorbers (such as CeO_2_ or ZnO) can represent a good solution for avoiding UV-related color changes in wood, especially the yellowing phenomenon (caused by the photodegradation of lignin and amorphous polysaccharides [7]) as proposed by Janesch et al. [65] and Weththimuni et al. [71]. Guo et al. [60] also demonstrated the application of TiO_2_/Ce nanomaterials, in the form of xerogels for the protection of Norway spruce wood samples against brown rot fungi (*Gloeophyllum* *trabeum*, *Rhodonia placenta*, and *Coniophora puteana*). The results revealed good antifungal protection but also the lack of any negative influence of the treatment on the mechanical properties (Brinell hardness test). The authors assigned the protective effect of the treatment to three main factors: (i) shielding of the cell wall by formation of a protective layer on the inner lumen surface; (ii) blocking of the micro/nanopores of the wood; (iii) a radical scavenging function.

**Figure 4:**
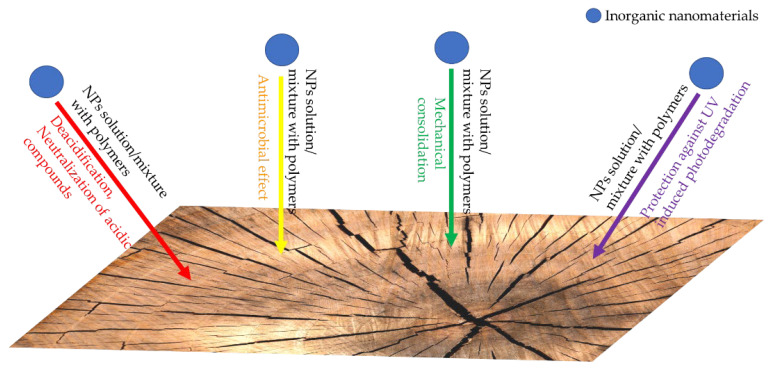
Potential application of nanomaterials for historical wood protection.

The most important applications of inorganic nanoparticles in the protection of historical wood are schematically presented in Figure 4.

## 5. Inorganic Nanomaterials for the Protection of Other Types of Cultural Heritage Artifacts of an Organic Nature

**Figure 5:**
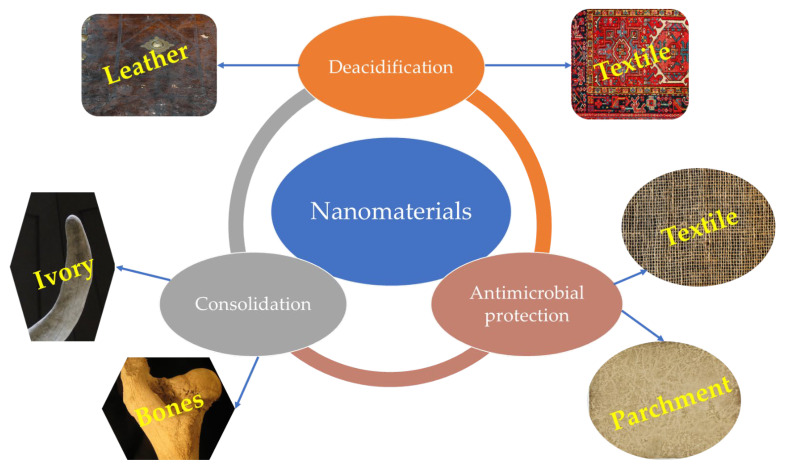
Examples of the nanomaterials’ applications in the conservation of other types of artifacts.

Although not as commonly encountered as in the case of paper and wood protection, nanomaterials also find application in the protection of other types of cultural heritage artifacts of organic nature. Representative examples for these applications are presented in Table 3 and Figure 5. In selecting the applications to be presented in Table 3, the origin of the artifacts was considered, not their present-day composition. As such, bones of archaeological interest or ivory pieces (known as ecofacts) were included in the literature review, although their present composition is mostly represented by the mineral phase.

As can be observed from Table 3, inorganic nanomaterials also find application in the protection of diverse cultural heritage artifacts such as dinosaur fossils, ivory, archaeological bones, leather bindings, parchment, or textiles. The consolidation application is correlated with the use of calcium-based nanomaterials (either calcium hydroxide or hydroxyapatite), as bone-type artifacts (ivory, bones, horns, antlers, etc.) have as a major inorganic constituent calcium phosphate. As such, their consolidation can be attempted using similar materials. Starting from these considerations, the in situ formation or the deposition of hydroxyapatite nanoparticles on artifacts with a high hydroxyapatite content represents an appropriate strategy. The materials are not only compatible with the inorganic component of the artifacts but are also able to fill the voids that appeared as a result of organic fraction destruction, increasing the mechanical properties of the treated artifacts and, consequently, their resistance. More than that, the application of the treatment does not negatively influence the possibility of recovering endogenous DNA molecules from the archaeological material [78].

Similar to the paper and wood applications, the deacidification of leather or dyed textiles can be achieved using similar compounds. The literature reviewed presented some examples on this topic, suggesting the application of calcium hydroxide or carbonate for deacidification purposes [73,79]. Their action on such artifacts is similar with the previously presented application, and particular attention is necessary for the interaction of the deacidification agent–collagen in the case of leather artifacts.

The final application identified in the literature, antimicrobial protection, is surprisingly underrepresented. Silver is a well-known antimicrobial agent, and its application was to be expected. However, the lack of studies regarding other well-known antimicrobial nanomaterials (such as copper nanoparticles, copper oxide, and zinc oxide) is equally surprising. The evaluation of phytosynthesized silver nanoparticles for the antimicrobial protection of parchment is remarkable [81]; however, in our opinion, this approach is highly underexplored. Their use could eliminate some of the risks associated with the application of silver nanoparticles including the risk of inducing esthetic alterations.

## 6. Conclusions and Future Perspectives

As a conclusion to the presented studies, inorganic nanomaterials represent a viable approach for the restoration and conservation of several types of cultural heritage artifacts of organic nature including paper, wood, papyrus, parchment, bone-type materials, leather, and textiles.

The application of nanomaterials can be divided in three main categories:

- Enhancement/protection of mechanical and esthetic characteristics;
- Protection from acid or UV-induced degradation (pH regulation, UV adsorption);
- Antimicrobial protection.

The selection of the materials for all these applications requires extensive studies before being possible to propose them at an industrial scale. Among the well-established applications, such as deacidification using calcium oxide or hydroxide, the potential improvements are related to the nanoparticles’ dimensions, which could influence the results of their application. Antimicrobial protection represents, in our opinion, the area with the most possibilities for improvement. For example, there could be studied other metallic or metal oxides nanoparticles phytosynthesized, a synthesis method which could enhance their properties and, at the same time, provide the appropriate manipulation of NP morphology [83]. Another prospective application is represented by nanomaterials with a double role. For example, calcium-substituted hydroxyapatite could be applied for the consolidation of artifacts, while an appropriate selection of the metal used for substitution (i.e., Zn, Co, or Ag) could also provide antimicrobial potential [84].

The main goal of cultural heritage protection should be the preservation of the artifacts in a state as close as possible to their original form as well as to offer the possibility to reverse the treatment, if necessary. Although promising, the application of nanomaterials for the protection of cultural heritage should be subjected to thorough tests in order to ensure that no detrimental effect is induced by the treatment, either mechanically or to the esthetic properties. Finally, another aspect that should be clarified in future studies is the possibility of establishing reversible treatments using nanomaterials, one of the major requests when discussing the protection of cultural heritage artifacts.

## Figures and Tables

**Table 1:** Application of inorganic nanomaterials for the treatment of historical paper artifacts (references presented in chronological order).

Nanomaterial Characteristics	Application Method	Obtained Results	References
MgO, nanorods (<100 nm)	Immersion of acidified paper in nanoparticle solution in propanol	Neutralization of a part of the sulfuric acid molecules. Treatment protected against hydrolytic degradation and depolymerization, regardless of the presence of gall ink. Deacidification led to an imperceptible increase in the brightness and yellowing.	[24]
Ca(OH)_2_, solvothermal method, dispersed in *n*-propanol in gelatin solution	Brush application of solution on paper containing gall ink (both simulated and real artifacts)	Simulated artifacts: significant differences in the cellulose degree of polymerization between treated and untreated samples were observed upon artificial aging. pH preservation at 9 after accelerated aging. Historical paper: pH preservation at 6.5.	[25]
ZnO, 150 nm, dispersed in ethanol with hydroxypropyl cellulose	Spray coating of different types of paper	Protection of paper against UV radiation, fungi, and bacteria.	[26]
Ca(OH)_2_, solvothermal method, dispersed in cyclohexane	Airbrush application on acidic paper and on historical paper artwork	Acidic paper: resilience to aging, lower cellulose depolymerization, and less color changes. Paper artwork: safeguarding of the original shape and topography of the support.	[27]
Ca(OH)_2_, dispersed in 2-propanol	Soaking in the NP solution	Stabilization of the pH values (7–8.2) 1 year after treatment and restoration of the alkaline reserve.	[28]
Ag, in combination with carboxymethyl cellulose, chitosan, soya beans flour, BEVA 371	Direct formation on the surface of the paper, followed by consolidant application on cotton linter and an 1887 book sample	Antimicrobial effect against *Staphylococcus aureus, Aspergillus niger, Candida albicans*, and *Pseudomonas aeruginosa* and improvement in the mechanical properties with a detrimental effect (color change) in combination with CMC and chitosan.	[29]
TiO_2_, 25 nm, dispersed in carboxymethyl cellulose with and without chitosan	Brush coating of Whatman filter paper	Developed adhesive led to protection against *Aspergillus flavus* and *A. niger*, increasing the tensile strength of the paper with a slight reduction in pH; protection against yellowing.	[30]
MgO–oleic acid in cyclohexane	Impregnation and immersion of different types of paper	Reduction in the surface pH of all types of acidic papers to ~8.0 without affecting the tensile strengths. After accelerated aging tests, surface pH and tensile strength values of treated samples were greater than the untreated ones; application in the inter-layer crevice and on the surface led to a change in the hydrophobicity of the papers (from hydrophilic to hydrophobic).	[31]
Mg(OH)_2_ (<100 nm) in trimethylsilyl cellulose	Dip coating of paper samples	The treatment reduced water wettability and increased mechanical strength of the paper. Mechanical properties (i.e., strength and elasticity) were improved with an increased number of coating steps	[32]
Mg(OH)_2_ (microwave-assisted synthesized nanosheets) in alcoholic aqueous solution	Air-spray coating of acidified and old paper samples	The treatment increased the pH value (from 2.5 to 10.5 for old paper), alkaline reserve (up to 372 mmol/kg for old paper), and mechanical properties (up to 6 GPa for Young’s modulus for old paper).	[33]
AgNPs (spherical, 7–13 nm) loaded in methyl methacrylate hydroxyethyl methacrylate	Brush coating on accelerated aged cotton and wood paper	Preservation of tensile strength and elongation rate upon accelerated aging.	[34]
ZnO, obtained by chemical route, with cellulose nanocrystals, 50 nm	In situ coating of newspaper	Increased color stability of the paper coated; antimicrobial effect of the nanocomposite against fungi (*A. niger, A. versicolor, Rhizopus nigricans, Saccharomycetes*, and *Mucor*) and bacteria (*Staphylococcus aureus* and *Escherichia coli*).	[35]
Biosynthesized spherical Ag (26–62 nm) and ZnO (8–23 nm)	Spray deposition of nanoparticles on paper inoculated with *A. niger* from old book paper	2 mM NP solutions prevented fungal biodeterioration and enhanced tensile strength.	[36]
MgO (sol-gel method), 12 nm	Impregnation of XVIIIth century paper with NP solution	10 mg/mL dispersion of MgO NPs provided complete inhibition of the *Trichoderma reesei, A. niger*, and *Cladosporium cladosporioides* fungal strains, avoiding color changes. Inhibited *A. niger* and *T. reesei* cellulase enzymes.	[37]
Biosynthesized Ag and ZnO nanoparticles	Deposition of nanoparticles on paper inoculated with *Bacillus subtilis* and *Penicllium chrysogenum* strains on a XVIIth century manuscript	1 mM AgNP and 2mM ZnO-NPs led to 100% microbial inhibition. Treated paper exhibited a slight color change and a similar structural analysis as the original paper.	[38]
MgO (50 nm) dispersed in hexamethyldisiloxane	Spray coating on 1954 wheat straw pulp paper, followed by the addition of saturated Ca(OH)_2_ solution	pH value increased to 7.5–9.0 and alkali storage to 220 mmol/kg. Tensile strength and folding degree increased by 28.05% and 80%. Color difference was negligible. The treatment provided good antimicrobial (*A. niger*) and anti-aging performances.	[39]
Mg_5_O(OH)_8_ lamellas, 5–10 nm thickness, obtained by pulsed laser ablation	80 year old paper washed in NP solution	Increased and stabilized the paper pH (no significant decrease during aging); enhanced the paper’s mechanical properties.	[40]
Ca(OH)_2_ (hexagonal, 60–90 nm) dispersed in in subcritical 1,1,1,2-tetrafluoroethane	Non-aqueous coating of naturally aged acidic paper including different pigments	The treatment neutralized the acidic functions of the papers, increased the alkaline reserve, and increased the mechanical strength, even compared to the classical spraying method.	[41]
CaCO_3_ (20 nm) alcoholic solutions, Ca(OH)_2_ commercial products	Immersion in nanoparticle solution followed by accelerated aging	All treatments led to alkaline pH values above 9 for commercial products and below 9 for carbonate; pH values should be under 9, as undesirable reactions could appear for lignocellulosic papers. Sufficient alkaline reserve (corresponding to the ISO/TS 18344/2016 standard). Less color change induced by carbonate compared with commercial products.	[42]
Nano-wollastonite (CaSiO_3_), 30–110 nm, in gel form, commercial	Impregnation of a 75 year old book	Significant hindering of *A. niger* growth at 20% and decreased permeability.	[43]
Ca(OH)_2_ (hexagonal platelets, 20–30 nm in thickness, 140 nm in diameter) and CaCO_3_ (70 nm) by solvothermal method	Brush coating of nanoparticles dispersed in oleic acid-grafted cellulose nanocrystals on acidified and aged paper	The treatment proved to be highly effective in the strengthening and deacidification of acidic and degraded paper, without significant alterations in the visual aspect of samples.	[44]
AgNPs (synthesized by chemical reduction, 8–10 nm)/nanocrystalline cellulose composites	Brush application on paper samples	The treatment enhanced the plastic properties of the paper, increasing inter-fiber interactions (leading to higher tensile strain resistance). Good biocidal activity against *A. niger*, while not affecting aesthetic appearance.	[45]
Na_2_CO_3_ solution and styrene acrylic latex composite	Ultrasonic atomization deposition on paper samples	Na_2_CO_3_ latex led to a pH higher than 7, only slight color change. Breaking length and the tear index were increased. Ink and handwriting were not diffused or smudged.	[46]
MgO in halloysite nanotubes in carboxymethyl cellulose	Impregnation of paper samples in nanocomposite solution	At 10% nanocomposite, treated paper retains a neutral pH value on exposure to acidic atmosphere. The tensile properties of the paper were improved after impregnation. Colorimetric properties and the writing quality were not modified after treatment.	[47]
AgNPs (10–80 nm) in aqueous solution	Disinfection in a misting chamber of photographic paper models	The AgNPs treatment proved to be less efficient disinfectant, compared with the ethylene oxide, against *Bacillus subtilis*, *Streptomyces* sp., *A. versicolor* and *T. viride.* AgNPs had less impact on the photographic models’ material properties (including color change and mechanical properties)	[48]

**Table 2:** Application of inorganic nanomaterials for the treatment of historical wood (references presented in chronological order).

Nanomaterial Characteristics	Application Method	Obtained Results	References
Ca(OH)_2_ hexagonal particles, 180 nm	Vacuum suction of NP solution into waterlogged archaeological wood (Vasa ship samples)	Deacidification of the treated samples, to neutral values, up to 8.	[54]
TiO_2_ (10–15 nm) and ZnO_2_ (10–30 nm) embedded in polyvinyl butyral	Treatment under vacuum of poplar wood	Antimicrobial protection against *Trametes versicolor* (white rot fungi) at 1% under light, color stability, and lignin degradation prevention.	[55]
SrCO_3_, 50 nm	Immersion and surface brushing of nanoparticle solution of oak waterlogged wood (Mary Rose ship)	Neutralization of sulfur-containing acidic compounds, with the formation of insoluble strontium sulfate; pH increased from 3 to up to 5.	[56]
ZnO, <100 nm, B_2_O_3_, <30 nm, CuO, 23–37 nm, TiO_2_, < 25 nm, CeO_2_, <25 nm, SnO_2_, <100 nm, commercial	Vacuum-treated sapwood portions of Scots pine, according to the BS EN 113 standard test method	CuO and SnO_2_ inhibited fungal decay by *T. versicolor* in weathered and unweathered specimens; all materials prevented decay by *Gloeophyllum trabeum* except for the B_2_O_3_-treated and weathered sample. CuO and B_2_O_3_ inhibited termite feeding. ZnO and CeO_2_ caused moderate termite resistance. ZnO and B_2_O_3_ inhibited mold growth.	[57]
Ca(OH)_2_ dispersed in ethanol (5 g/L)	Direct dipping of nanocomposite solution for consolidation of waterlogged archaeological wood (softwood and hardwood), alum-treated archaeological wood (Oseberg find), and sound oak	pH increased (2–3 units); on degraded samples with very small amounts of cellulose, a pH of 5.5 was reached; stabilization after 1 month.	[58]
ZnO, <100 nm, B_2_O_3_, <30 nm, CuO, 23–37 nm, TiO_2_, <25 nm, CeO_2_, <25 nm, SnO_2_, <100 nm, commercial, combined with Paraloid B72 consolidant	Vacuum-treated sapwood portions of Scots pine, according to the BS EN 113 standard test method	Nanomaterials used in combination with the consolidant slowed fungal degradation and did not inhibit surface mold growth; improved water resistance. Consolidant treatment reduced nanoparticle leaching.	[59]
TiO_2_/Ce xerogel	Soaking of Norway spruce wood followed by evaluation of antifungal efficiency (*G. trabeum*, *Rhodonia placenta*, and *Coniophora puteana*, acc. EN 113 standard) and mechanical assays	The treatment led to increased resistance against brown rot decay, maintaining the mechanical properties.	[60]
Ca(OH)_2_ encapsulated in halloysite nanotubes in PEG 1500 solution	Immersion in nanocomposite solution for consolidation of waterlogged archaeological wood (Chretienne C ship)	Mechanical consolidation (increase in the elastic modulus and stress at the breaking point) and deacidification of the treated samples (pH = 7.6 12 months after treatment).	[61]
AuNPs (50 nm)/hydroxyapatite composites	Brushing of young and aged hazelnut wood	Stopping the wood weathering process (increased surface hardness, increased hydroscopic stability).	[62]
ZnO rod-shaped particles obtained by microwave-assisted solvothermal method	Impregnation of pine tree samples	Improved pinewood decay resistance against white rot fungi (*Ganoderma applanatum*) above 2.5% content without affecting the hardness results.	[63]
ZnO, 29 nm, in polyvinyl butyral matrix	Immersion on consolidant solution of historic wood and oriental plane samples	ZnO addition lowered the degradation rate at accelerated aging and decreased water penetration and wettability. Optimum concentration was 1 wt% ZnO.	[64]
CeO_2_ embedded in biopolymers (chitosan or cationic starch)	Immersion in NP/biopolymer solution of spruce wood	Reducing UV-related color changes (especially yellowing).	[65]
ZnO_2_ (spherical, 8–15 nm, solvothermal method) and TiO_2_ (under 25 nm, hydrothermal method) embedded in polymer	Immersion in NP/polymer solutions of cedar and sycamore woods	Increased mechanical properties (increased bending and compression resistances).	[66]
Ca(OH)_2_—hexagonal lamellas, 10 nm, Mg(OH)_2_—hexagonal lamellas, under 10 nm	Immersion on NP solution for preventive and curative treatment of waterlogged wood (Gallo–Roman wreck)	Deacidification of the treated samples to neutral pH values.	[67]
Al_2_(SO_4_)_3_, CuSO_4_·5H_2_O, H_3_BO_3_ introduced into H_3_PO_4_	Treatment of sapwood	Efficient mildew resistance after 28 days of exposure to *A. niger* and *T. viride*.	[68]
Halloysite nanotubes/pluronic nanocomposites	Treatment of waterlogged wood	Nanotubes added reaching the internal part of the wood (consolidating not only the surface) through the lignin channels.	[69]
Halloysite nanotubes in molten paraffin wax	Immersion on composite solution of waterlogged wood	Overall, improvement in the mechanical properties; Young’s modulus/stress at breaking increased, the elongation at break decreased, in the absence of side effects.	[70]
ZnO (30–110 nm), ZrO_2_ (90-230 nm), functionalized with 3-glycidoxypropyltrimethoxysilane (GPTMS) in shellac-based varnishes	Brushing of maple wood samples	Increased resistance to alcoholic media; no negative effect on the chromatic properties of the coating; improved water-repellence behavior; ZrO_2_ varnish increased resistance to scratches; ZnO varnish increased resistance to UV aging and enhanced resistance to mold growth.	[71]

**Table 3:** Application of inorganic nanomaterials for the treatment of other types of cultural heritage of an organic nature (references presented in chronological order).

Artifact Type	Nanomaterial Characteristics	Application Method	Obtained Results	References
Pre-Columbian archaeological textiles	AgNPs, 10–15 nm and 50–80 nm	Applied using a patented method to a concentration of 4.5 ppm/g of textile/disinfection cycle	Application of AgNPs led to the protection of textiles against *Pseudomonas aeruginosa* (by 63–97%, depending on the strain and exposition time).	[72]
Historical leather (XVIIIth century)	Ca(OH)_2_, solvothermal method, hexagonal platelets, 20–30 nm (thickness)	Mixed with calcium lactate nanoparticles, applied by dipping on real and historical leather	After treatment application, the pH of the historical leather was adjusted to 4.5 with no detrimental effect on the collagen.	[73]
Pre-Columbian archaeological textiles	AgNPs (10–80 nm)	Misting disinfection using patented installation	The treatment led to a reduction in microbial contamination by 30.8–99.9% (depending on the microbial species and initial level of contamination) for several microbial lines (most resistant—*Bacillus* spp.; more sensitive—*Oceanobacillus*, *Kocuria*, *Paracoccus*, *Cladosporium*, and *Penicillium* spp.) No changes were recorded in the pH values and esthetic characteristics of the treated samples.	[74]
Linen fabric samples (simulating old stained samples)	TiO_2_, ZnO, 3–18 nm, commercial	Spraying the simulated old stained samples	TiO_2_ treatment led to higher fading of the stains, compared with ZnO; TiO_2_ had higher hydrophobicity than ZnO. Due to the fact of safety reasons, to protect the artifacts, the authors suggest the use of ZnO as self-cleaning agent.	[75]
Cotton exposed to fungi commonly found on ancient textiles	Wollastonite (CaSiO_3_) nanofibers, 30–110 nm, commercial	Immersion of cotton strips in nanomaterial gel (20%)	Impregnation led to significant limitation of *A. niger* activity on cotton as demonstrated by the tensile tests.	[76]
Ivory (from ancient elephant tusks)	Hydroxyapatite (HAP): spherical, 50 nm, hydrothermal method	Immersion in the colloidal solution, dried	After treatment, an HAP layer formed (protecting the ivory from further deterioration), repairing the loose and porous surface. Hardness, elastic modulus, and anti-scratch performance were significantly improved. No esthetic changes were recorded.	[77]
Archaeological human bone remains, Iron age	Ca(OH)_2_ nanoparticles dispersed in 2-propanol and diammonium hydrogen phosphate (DAP)	Bones soaked for in Ca(OH)_2_, dried, soaked in DAP	In situ synthesis of hydroxyapatite, led to an increase in hardness (up to 56%) and mineral density, and there was a significant reduction in pore volume and surface area. No substantial effect on the ability to recover endogenous DNA molecules was recorded.	[78]
Iron tannate dyed cotton	CaCO_3_ (90 nm), SiO_2_ (spherical, 35 nm), in diverse complex combination with polyethyleneimine, carboxymethylcellulose, cellulose nanofibers, or polyvinylpyrrolidone	Nebulization/brush application on naturally and accelerated aged samples	SiO_2_ nanoparticles, in combination with Nanocellulose, stabilized the naturally aged samples, while calcium carbonate nanoparticles were used as deacidification treatment (pH changed from 3.6 to 7.5). CaCO_3_ also protected strengthening agents, which led to an increase in the mechanical properties of the samples; after artificial aging, the deacidified samples revealed a slowing down of cotton degradation.	[79]
Pterosaur fossils	Ca(OH)_2_ (hexagonal, 20 nm)/kaolin (nanosheets, 4–12 nm thickness) nanocomposite, dispersed in ethanol	Brush application to saturation	The treatment had no significant effect on the breathability of the fossil, significantly enhanced the consolidation strength of the fossil, porosity was reduced to 51%; no eye-detected effects on the color of the fossil.	[80]
Parchment from goat skin	Tea leaf-mediated AgNPs, spherical, oval, and hexagonal shapes, 20–50 nm	Deposited on parchment samples, artificially aged	Antimicrobial treatment effective against bacteria and fungi (*Streptomyces* *Albidoflavus*, *Cladosporium xanthochromaticum, A. fumigatus*, *Byssochlamys spectabilis*) at a 0.025% concentration. The treatment did not significantly influence the chemical and mechanical characteristics of treated parchment even after accelerated thermal aging.	[81]
Simulated bone artifacts	Ca(OH)_2_, 217 nm, dispersed in 2-propanol	In situ growth of Ca(SO_4_)_2_ by the drip-permeance method	A Ca(SO_4_)_2_·2H_2_O continuous phase formed in situ which filled the holes, bridged the cracks, and conferred strength to the bones, maintaining their original appearance. Microhardness increased by 3 times, porosity reduced by 10%, and the color difference by 2.7.	[82]

## Data Availability

Not applicable.
